# Hyperprolactinemia in Children with Subclinical Hypothyroidism

**DOI:** 10.4274/jcrpe.4536

**Published:** 2017-12-15

**Authors:** Neera Sharma, Deep Dutta, Lokesh Kumar Sharma

**Affiliations:** 1 Dr. Ram Manohar Lohia Hospital and Post Graduate Institute of Medical Education and Research, Department of Biochemistry, New Delhi, India; 2 Venkateshwar Hospital, Clinic of Diabetes, Endocrinology and Metabolic Disorder, New Delhi, India; 3 Dr. Ram Manohar Lohia Hospital and Post Graduate Institute of Medical Education and Research, Department of Endocrinology, New Delhi, India

**Keywords:** Subclinical hypothyroidism, hyperprolactinemia, prolactin, thyroid stimulating hormone

## Abstract

Prevalence of hyperprolactinemia in children with subclinical hypothyroidism (ScH) is not known. This study aimed to determine the occurrence and predictors of hyperprolactinemia in euthyroid children and in children with ScH and overt primary hypothyroidism (OPH). Serum prolactin levels were estimated in consecutive children <18 years of age undergoing thyroid function evaluation and diagnosed to have normal thyroid function, ScH, or OPH. Children with pituitary adenomas, secondary hypothyroidism, multiple pituitary hormone deficiency, comorbid states, and drug-induced hyperprolactinemia were excluded. From the initially screened 791 children, hormonal data from 602 children who fulfilled all criteria were analyzed. Seventy-one (11.79%) of these had ScH, and 33 (5.48%) had OPH. Occurrence of hyperprolactinemia was highest in the OPH group (51.51%), followed by ScH (30.98%) and euthyroid children (4.41%) (p<0.001). Median (25th–75th percentiles) levels for prolactin in euthyroid, ScH, and OPH children were 13.3 (9.4-17.95), 19.15 (15.97-30.12), and 28.86 (17.05-51.9) ng/mL, respectively (p<0.001). In children, prolactin levels were comparable in males and females. An age-related increase in serum prolactin was noted in euthyroid children, which was statistically significant in post-pubertal (16-18 years) children. Area under the curve for thyroid stimulating hormone (TSH) in predicting hyperprolactinemia in children was 0.758 (95% confidence interval: 0.673–0.829; p<0.001). TSH ≥4.00 mIU/L had a sensitivity of 69.4% and specificity of 77.6% in detecting hyperprolactinemia. Hyperprolactinemia is common in children with ScH and OPH. TSH ≥4.00 mIU/L has a good sensitivity and specificity in predicting hyperprolactinemia in children. More studies are needed to establish if hyperprolactinemia should be an indication for treating ScH in children.

**What is already known on this topic?**

Hyperprolactinemia is common in adults with subclinical and overt primary hypothyroidism. Hyperprolactinemia in children has been linked to adverse metabolic outcomes. Prevalence of hyperprolactinemia in children with thyroid dysfunction, especially subclinical hypothyroidism, is not known.

**What this study adds?**

Hyperprolactinemia is common in children with hypothyroidism, observed in a third of children with subclinical hypothyroidism and more than half of children with overt hypothyroidism. Receiver operating characteristics analysis showed that thyroid-stimulating hormone ≥4.00 mIU/L has a good sensitivity and specificity in predicting hyperprolactinemia in children. Hyperprolactinemia may be an indication for treating subclinical hypothyroidism in children.

## INTRODUCTION

Hyperprolactinemia is a common endocrinopathy encountered in clinical practice with a prevalence ranging from 0.4% to 5% (1). We recently reported that in a cohort of 2848 individuals, hyperprolactinemia was common in patients with subclinical hypothyroidism (ScH), especially in those with thyroid stimulating hormone (TSH) >7.5 mIU/L (2). TSH ≥7.51 mIU/L (females) and ≥8.33 mIU/L (males) had a sensitivity of ≈50% with a high specificity of >90% in detecting hyperprolactinemia (2). Overt hypothyroidism in children has been linked with pituitary enlargement, pituitary hyperplasia, and in rare cases, even with feedback pituitary adenoma and multiple pituitary hormone deficiency (3,4). This enlargement of the pituitary is primarily believed to be due to increase in the number and size of thyrotrophs and lactotrophs in the pituitary gland (3). Primary hypothyroidism leads to increased thyrotropin-releasing hormone (TRH) release from hypothalamus, which has a physiologic trophic effect on thyrotrophs and lactotrophs resulting in increased TSH and prolactin levels, respectively (3,5). Reduced prolactin clearance as well as reduced sensitivity to the inhibitory actions of dopamine and its agonists on prolactin release also contribute to hyperprolactinemia (6). However, prolactin levels have rarely been investigated in children with hypothyroidism, especially in those with ScH. Also, the prevalence of hyperprolactinemia in children with ScH is not known. Hence, the aim of this study was to determine the occurrence and predictors of hyperprolactinemia in children with a spectrum of thyroid dysfunction ranging from euthyroidism to ScH and overt primary hypothyroidism.

## METHODS

Consecutive children younger than 18 years undergoing thyroid function evaluation at the department of biochemistry were selected for investigation. Children diagnosed to have pituitary adenomas, secondary hypothyroidism, multiple pituitary hormone deficiency, subclinical hyperthyroidism, and overt hyperthyroidism were excluded. Also, children with associated comorbidities such as chronic liver disease, renal disease, syndromes, and those taking medications such as anti-epileptics, neuroleptics, and anti-psychotics were excluded. The study protocol was explained, and only those children or their parents/guardians who gave informed written consent were included in the study. Serum prolactin was assayed from samples of children undergoing thyroid function evaluation who fulfilled the above criteria and who were diagnosed to have either ScH, overt primary hypothyroidism, or normal thyroid function. The reference range of free tri-iodothyronine (FT3), free tetra-iodothyronine (FT4), and TSH in our laboratory is 2–4.4 pg/mL, 0.6–2.2 ng/dL, and 0.5–5 mIU/L, respectively. ScH was defined as normal FT4 levels with TSH levels above the normal range (7). Overt primary hypothyroidism was defined as TSH levels above the normal range accompanied by low FT4 levels (7). Serum was separated from samples collected and stored at −80 °C. Patients with drug-induced hyperprolactinemia were excluded. The study duration was from August 2014 to December 2016. The Institutional Ethics Committee of Dr. Ram Manohar Lohia Hospital and Post Graduate Institute of Medical Education and Research approved the study [approval number: 89 (13/2014/IEC/PGIMER/RML/1645) dated 11th June 2014]. Serum prolactin was measured using chemiluminescence microparticle immunoassay (CLIA) (VITROS® ECiQ Immunodiagnostic System, Johnson & Johnson, USA), a methodology which has been elaborated elsewhere (2). The normal ranges of serum prolactin in adults are 2.8–27 ng/mL in females and 2.1–17 ng/mL in males (2). We used the age- and sex-specific normal ranges of serum prolactin in children developed by Aitkenhead and Heales (8) for defining hyperprolactinemia in our study. There is no sex difference in serum prolactin among children till 1 year of age. All children 16 years old or younger, with serum prolactin levels higher than the upper limit (97th percentile) for their age and sex were defined to have hyperprolactinemia (Table 1). For children between 16 and 18 years of age, the adult reference range was used in the analysis. Hence, hyperprolactinemia among children in the 16-18 years old group was defined as a serum prolactin level >27 ng/mL in females and >17 ng/mL in males (2).

The VITROS® ECiQ Immunodiagnostic System, Johnson & Johnson, USA was also used for estimation of FT3, FT4, and TSH. The methodology has been elaborated elsewhere (9). Samples from children having elevated prolactin levels with normal thyroid function were evaluated for macroprolactinemia using polyethylene glycol (PEG) precipitation test (treatment of equal parts of serum with PEG followed by centrifugation) to remove macroprolactin. Macroprolactinemia was diagnosed if post-PEG prolactin level was <40% of pre-PEG levels (10).

### Statistical Analysis

Normality of the distribution of variables was assessed using the Kolmogorov-Smirnov test, and accordingly parametric or non-parametric tests were used for statistical analysis. The receiver operating characteristics (ROC) curves were plotted, and areas under the curves (AUCs) with 95% confidence interval (CI) were calculated to explore the diagnostic efficacy and determine cut-offs of serum TSH in predicting hyperprolactinemia in children. The Youden index, defined as (sensitivity + specificity) −1 was used to determine the optimal cut-off points. A p-value <0.05 was considered statistically significant. SPSS version 20 was used for the analyses.

## RESULTS

Of a total of 791 children younger than 18 years of age who underwent thyroid function evaluation, 649 (males:females=200:402) who fulfilled all inclusion and exclusion criteria had their serum prolactin levels determined. Drug-induced hyperprolactinemia was diagnosed in 47 children who were excluded. Other reasons for exclusion were secondary hypothyroidism (n=23), multiple pituitary hormone deficiency (n=16), associated pituitary adenoma (n=12), comorbid disease states (n=85), and refusal to consent (n=6). Hence, 602 children were evaluated in the study. Of these, 71 children (11.79%) were diagnosed to have ScH and 33 (5.48%) had overt primary hypothyroidism. The remaining 498 children were euthyroid. The occurrence of hyperprolactinemia in subclinically hypothyroid and overt hypothyroid children was 22 (30.98%) and 17 (51.51%), respectively, figures which were significantly higher as compared to euthyroid children (4.41%) (p<0.001). Among the 71 children with ScH, the occurrence of hyperprolactinemia in children with TSH levels of 5-7.5, 7.5-10, and >10 mIU/L was 23.80% (15/63), 80% (4/5), and 100% (3/3), respectively (p<0.001). Median (25th–75th percentile) values for serum prolactin levels in euthyroid children, children with ScH, and those with overt primary hypothyroidism were 13.3 (9.4-17.95) ng/mL, 19.15 (15.97-30.12) ng/mL, and 28.86 (17.05-51.9) ng/mL, respectively (p<0.001).

**Figure 1:**
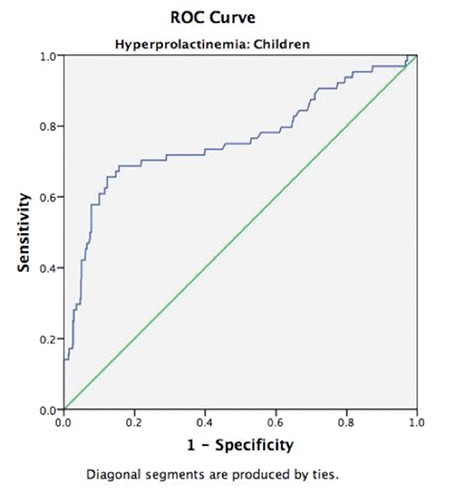
Receiver operating characteristics curve (blue line) showing the sensitivity and specificity of serum thyroid stimulating hormone in predicting hyperprolactinemia in children ROC: receiver operating characteristics

Among the 602 children evaluated in this study, 33 were in age group 0-5 years, 54 in age group 6-10 years, 211 in age group 11-15 years, and 304 in age group 16-18 years (Table 2). Median prolactin levels among children in the different age groups (euthyroid vs. hypothyroidism) are shown in Table 3. As shown in Table 3, serum prolactin levels increased with age in euthyroid children, an increase which was significantly higher in the post-pubertal (16-18 years) age group (p<0.001). A similar trend was not seen in children with hypothyroidism (Table 3). Median prolactin levels of children in the different age groups were not significantly different based on the sex of the child (Table 4). The areas under the ROC curves were constructed to evaluate the predictive values of serum TSH in predicting hyperprolactinemia in children. The AUC for TSH in predicting hyperprolactinemia in children was 0.758 (95% CI: 0.673–0.829; p<0.001). A serum TSH ≥4.00 mIU/L had a sensitivity of 69.4% and specificity of 77.6% in detecting hyperprolactinemia (Figure 1).

## DISCUSSION

Undiagnosed and untreated hyperprolactinemia has been linked with poor bone mineral health, osteomalacia, hypogonadotropic hypogonadism, menstrual abnormalities, and ovulatory dysfunction in adults (11,12). 

Hyperprolactinemia is believed to be rare in children (13). Drug-induced hyperprolactinemia secondary to anti-epileptics, antipsychotics, and hyperprolactinemia secondary to pituitary tumors are believed to be the most common causes of hyperprolactinemia in children (14,15). Hyperprolactinemia in children has also been linked with impaired bone health, puberty problems, galactorrhea in females, and gynecomastia in males (16,17).

Our study highlighted for the first time that hyperprolactinemia is common in children with ScH (30.98%). The occurrence of hyperprolactinemia in children with overt hypothyroidism in this study was 51.51% and its occurrence in euthyroid children was 4.41%. These trends are similar to those observed in adults. We have previously reported the occurrence of hyperprolactinemia in adults with euthyroidism, ScH, and overt hypothyroidism to be 2.02-2.32%, 31.61-35.65%, and 39.53-42.95%, respectively (2).

An important observation of this study was that in contrast to adults (females have higher prolactin levels than males), no difference in prolactin levels was noted between male and female children. Prolactin levels in post-pubertal children (16-18 years of age) were significantly higher compared to prepubertal (0-5 years, 6-10 years) and peri-pubertal children (11-15 years). Increased sex steroid levels in the post-pubertal state, especially estrogen, may explain this finding. The trophic effect of estrogen on TRH-mediated prolactin secretion from lactotrophs has been reported in various studies (18).

A serum TSH level ≥4.00 mIU/L had a sensitivity and specificity of 69.4% and 77.6%, respectively in detecting hyperprolactinemia in children. We have previously demonstrated that TSH levels of ≥7.51 mIU/L in females and ≥8.33 mIU/L in males can be used as cut-off levels in detecting hyperprolactinemia in adults (2). There is often a lack of clarity among doctors with regard to indications for levothyroxine therapy in ScH (19). Hyperprolactinemia may be accepted as one of the indications for levothyroxine therapy in adults with ScH (2).

This study highlighted that the TSH thresholds for detecting hyperprolactinemia is much lower in children as compared to adults (4 mIU/L vs. 7.5 mIU/L). This statement is also supported by the observation that in our study, all children with ScH who had TSH levels ≥7.51 mIU/L had hyperprolactinemia as compared to only 49-61% of adults reported in a previous study (2). The limitations of this study include the lack of assessment of gonadotropins and sex steroids in children in different age groups and their relationship with prolactin and thyroid hormone levels. Obesity was also not assessed among children in our study cohort and serum prolactin levels were not adjusted for childhood obesity. There is a report suggesting that serum prolactin may be lower in children with obesity (20). However, it must be highlighted that our institute is a government medical college and hospital, providing free medical treatment to the population. The majority of our patients are the off spring of parents of low socio-economic status, and obesity is rare in these children. Being a cross-sectional study, the impact of levothyroxine supplementation on prolactin levels in ScH and overt primary hypothyroidism was not evaluated, and is a limitation.

To conclude, it may be said that hyperprolactinemia is common in children with hypothyroidism, observed in a third of children with ScH and in more than half of children with overt hypothyroidism. ROC analysis confirmed that a TSH ≥4.00 mIU/L has a good sensitivity and specificity in predicting hyperprolactinemia in children. Further longitudinal studies are warranted to evaluate the impact of this hyperprolactinemia on clinical outcomes in children and to establish if hyperprolactinemia should be considered as an indication for treating ScH in children.

## Figures and Tables

**Table 1:**
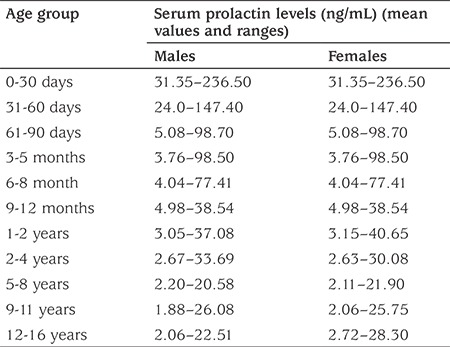
Normal levels (ranging from 2.5th and 97.5^th^ percentiles) for serum prolactin in children of different age groups

**Table 2:**
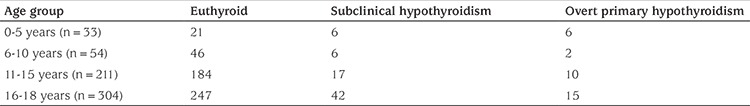
Occurrence of subclinical and overt primary hypothyroidism in children of different age groups in this study

**Table 3:**
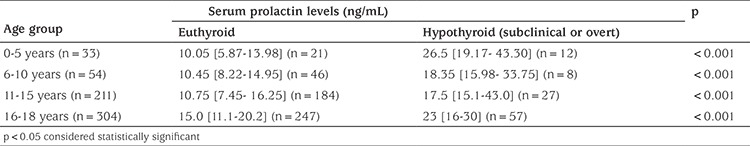
Serum prolactin levels in euthyroid children as compared to those with subclinical or overt hypothyroidism in different age groups (median and 25^th^–75^th^ percentile values)

**Table 4:**
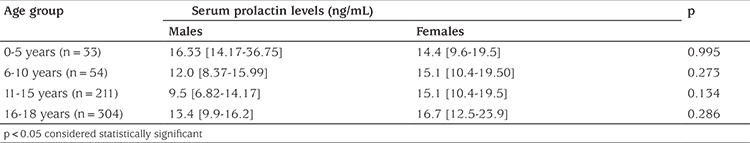
Median prolactin levels in male and female children of different age groups (median and 25^th^–75^th^ percentile values)
